# Alteration of Colonic Mucosal Permeability during Antibiotic-Induced Dysbiosis

**DOI:** 10.3390/ijms21176108

**Published:** 2020-08-25

**Authors:** Ying Ran, Hirokazu Fukui, Xin Xu, Xuan Wang, Nobuhiko Ebisutani, Yoshiki Tanaka, Ayako Maeda, Yutaka Makizaki, Hiroshi Ohno, Takashi Kondo, Tomoaki Kono, Katsuyuki Tozawa, Toshihiko Tomita, Tadayuki Oshima, Hiroto Miwa

**Affiliations:** 1 Division of Gastroenterology and Hepatology, Department of Internal Medicine, Hyogo College of Medicine, 1-1, Mukogawa, Nishinomiya 663-8501, Japan; ranying1988@126.com (Y.R.); onlyxx1984@163.com (X.X.); a43onok@126.com (X.W.); no-ebisutani@hyo-med.ac.jp (N.E.); kondou@hyo-med.ac.jp (T.K.); kono@hyo-med.ac.jp (T.K.); katu-you@hyo-med.ac.jp (K.T.); tomita@hyo-med.ac.jp (T.T.); t-oshima@hyo-med.ac.jp (T.O.); miwahgi@hyo-med.ac.jp (H.M.); 2 Department of Gastroenterology and Hepatology, Tianjin Medical University General Hospital, Anshan Road 154, Heping District, Tianjin 300052, China; 3 R&D Center, Biofermin Pharmaceutical Co., Ltd., 7-3-4, Higashi-machi, Ibukidai, Nishi-ku, Kobe 651-2242, Japan; tanaka_yoshiki@biofermin.co.jp (Y.T.); maeda_ayako@biofermin.co.jp (A.M.); makizaki_yutaka@biofermin.co.jp (Y.M.); ohno_hiroshi@biofermin.co.jp (H.O.)

**Keywords:** dysbiosis, antibiotic, permeability, cytokine, tight junction, gut microbiome, claudin, colorectal, vancomycin, polymyxin B

## Abstract

Although dysbiosis is likely to disturb the mucosal barrier system, the mechanism involved has remained unclear. Here, we investigated alterations of colonic mucosal permeability and tight junction (TJ) molecules in mice with antibiotic-induced dysbiosis. Mice were orally administered vancomycin or polymyxin B for 7 days, and then fecal samples were subjected to microbial 16S rRNA analysis. The colonic mucosal permeability was evaluated by chamber assay. The colonic expression of TJ molecules and cytokines was examined by real-time RT-PCR, Western blotting, and immunohistochemistry. Caco2 cells were stimulated with cytokines and their transepithelial electric resistance (TEER) was measured. Vancomycin-treated mice showed significantly lower gut microbiota diversity than controls, and the same tendency was evident in polymyxin B-treated mice. The colonic mucosal permeability was significantly elevated in both vancomycin- and polymyxin B-treated mice. The expression of claudin 4 in the colonic mucosa was decreased in both vancomycin- and polymyxin B-treated mice. Colonic expression of *TNF-α* and/or *IFN-γ* was significantly increased in mice that had been administered antibiotics. TNF-α and IFN-γ stimulation dose-dependently decreased TEER in Caco2 cells. Antibiotic-induced dysbiosis is correlated with the enhancement in colonic tissue permeability, accompanied by a reduction in claudin 4 expression and enhancement in *TNF-α* and/or *IFN-γ* expression in mice.

## 1. Introduction

The intestinal mucosal barrier system is crucial for preventing the invasion of pathogens into the living host [1]. Tight junctions (TJs) are the most important structural components responsible for constitutive barrier function in epithelial cells [2]. However, other factors such as mucins, antimicrobial molecules, cytokines, and immunoglobulins also play a role in the maintenance of the intestinal mucosal barrier system [3]. All of these factors are well-orchestrated in the gastrointestinal tract under symbiotic conditions, facilitating normal intestinal permeability [4]. However, once intestinal permeability has increased, bacterial components such as lipopolysaccharide easily pass the mucosal barrier, resulting in the development of inflammation [5]. Recently, the disruption of mucosal barrier “leaky gut” has been highlighted as this pathophysiology is key not only in functional gastrointestinal disorders but also in metabolic and psychological diseases [6,7]. However, it is unclear what factors play a crucial role in the regulation of intestinal permeability.

Accumulating evidence has recently revealed that an imbalance in the gut microbiota profile (dysbiosis) is importantly involved in the pathophysiology of various diseases in the host [8]. Of note, the gut microbiota is considered to impact considerably on intestinal barrier function through direct interaction with epithelial cells or indirect stimulation through production of metabolites such as short-chain fatty acids (SCFAs) [9]. In this context, dysbiosis is likely to disturb the mucosal barrier system [10]. However, it remains unclear how dysbiosis affects mucosal barrier function in the intestinal tract.

To investigate the effect of dysbiosis on mucosal barrier function, we prepared a mice model by treatment with the antibiotics vancomycin and polymyxin B, which kill mainly Gram-positive and -negative bacteria, respectively. Thus, we initially expected that different antibiotic treatments may have different effects on gut flora, intestinal permeability, and mucosal immunity. In the present study, we then investigated alterations in the gut microbiota profile and colonic mucosal permeability in these mice with antibiotic-induced dysbiosis. Furthermore, we examined the expression of TJ molecules and cytokines in the colon while confirming the effect of specific cytokines on epithelial barrier function.

## 2. Results

### 2.1. Effect of Antibiotic Treatment on Mouse Gut Microbiota Structure

**Figure 1:**
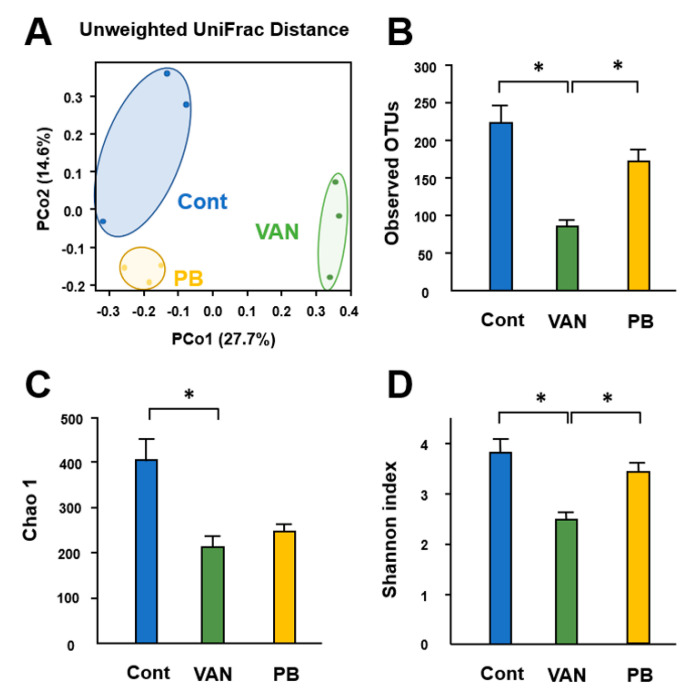
Effect of treatment with vancomycin or polymyxin B on gut microbiota. (**A**) Unweighted UniFrac principal coordinate analyses (PCoA) showing clustered communities of intestinal microbiota in the experimental mice. PCo1 and PCo2 describe the indicated percentage of variation on the x-axes and y-axes, respectively. (**B**) Analysis of operational taxonomic units (OTUs). Alpha-diversity of gut microbiota (**C**, Chao1; **D**, Shannon Index). Results are expressed as the mean ± SE (n = 3 in each group). Significant differences between two groups at * *p* < 0.05. Significance of differences was determined by Welch’s *t*-test with Benjamini–Hochberg correction. Cont, control; VAN, vancomycin; PB, polymyxin B.

To confirm whether vancomycin or polymyxin B treatment caused dysbiosis in the experimental mice, we analyzed alterations in the gut microbiota profile. Unweighted UniFrac-based principal coordinate analysis (PCoA) showed differential clustering of the gut microbiota structures among the control, vancomycin-treated, and polymyxin B-treated mice (Figure 1A). This suggested that not only did vancomycin and polymyxin B cause dysbiosis, but the type of dysbiosis also differed between vancomycin- and polymyxin B-treated mice. The number of observed operational taxonomic units (OTUs) in vancomycin-treated mice was lower than in the controls (*p* < 0.05) and the polymyxin B group (*p* < 0.05). In addition, the number of observed OTUs tended to be lower in polymyxin B-treated mice (*p* = 0.137) than in the controls (Figure 1B). The alpha-diversity of fecal microbiota was evaluated in terms of the Chao1 and Shannon indices. The Chao1 index was significantly lower in vancomycin-treated mice (*p* < 0.05), and the same tendency was observed in polymyxin B-treated mice (*p* = 0.052) (Figure 1C). The Shannon index was significantly lower in vancomycin-treated mice than in both control and polymyxin B-treated mice (*p* < 0.05) (Figure 1D).

**Figure 2:**
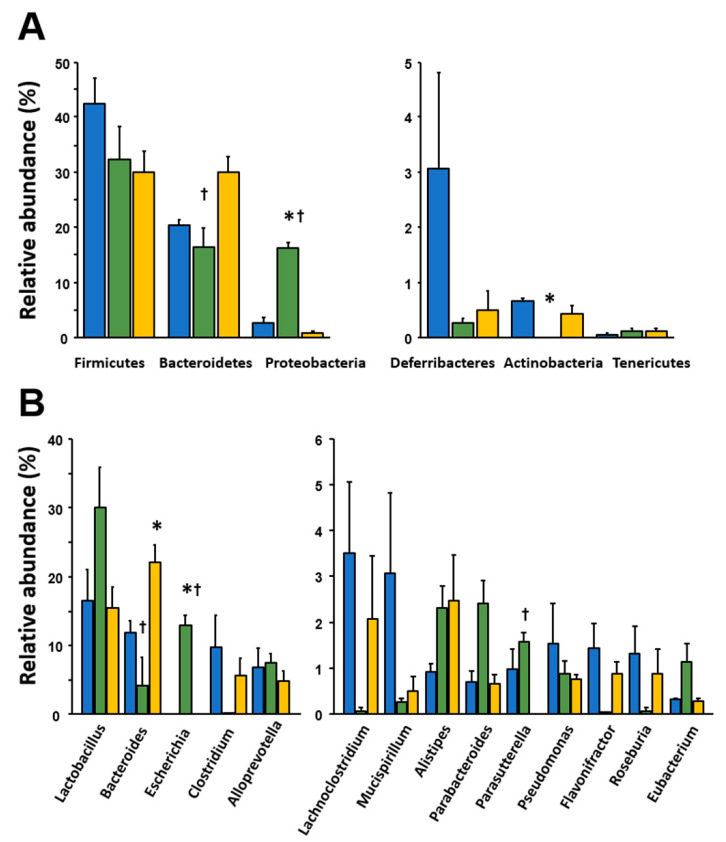
Effect of antibiotic treatment on the relative abundance of intestinal bacteria at (**A**) the phylum and (**B**) the genus level. The relative abundance of each bacterial phylum and genus was analyzed by next-generation sequencing of bacterial 16S rDNA. Cont, control (blue); VAN, vancomycin (green); PB, polymyxin B (yellow). Results are expressed as the mean ± SE (n = 3 in each group). Significant differences between the control and antibiotic-treated groups at * *p* < 0.05 or between the vancomycin- and polymyxin B-treated groups at † *p* < 0.05. Statistical significance was determined by Welch’s t-test with Benjamini–Hochberg correction.

Moreover, we examined the phylum and genus profiles of gut microbiota in the experimental mice. Among the six major phyla, Proteobacteria were markedly more abundant (*p* < 0.01) and Actinobacteria were significantly less abundant (*p* < 0.01) in vancomycin-treated mice than in the controls; there were no significant changes in these phyla in polymyxin B-treated mice (Figure 2A). The abundance of Bacteroidetes tended to increase in polymyxin B-treated mice relative to the controls (*p* = 0.06) and was significantly higher in polymyxin B-treated than in vancomycin-treated mice (*p* < 0.05).

At the genus level, we further found that Escherichia and Bacteroides were significantly more abundant in vancomycin- and polymyxin B-treated mice than in the controls, respectively (Figure 2B). When vancomycin- and polymyxin B-treated mice were compared, Escherichia and Parabacteroides were significantly more abundant in the former, whereas Bacteroides tended to be more abundant in the latter.

### 2.2. Effect of Antibiotic Treatment on Mouse Colonic Physiology

**Figure 3:**
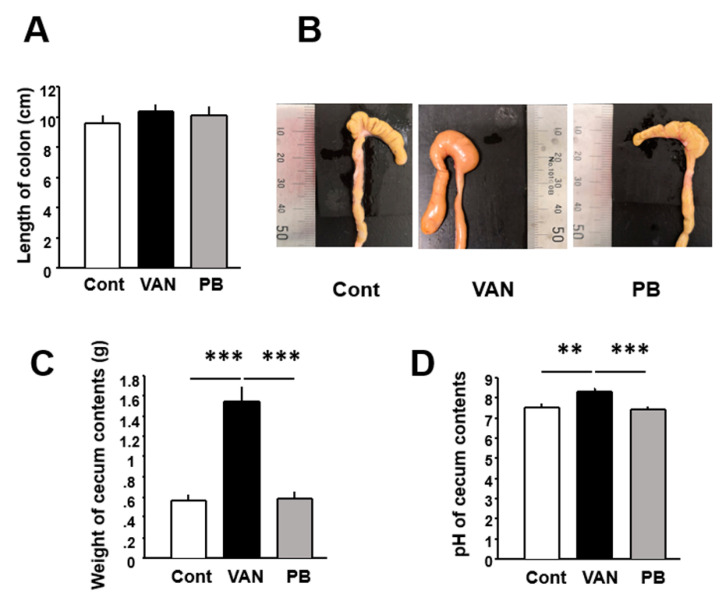
Effect of antibiotic treatment on colon length and cecum contents. (**A**) Length of the colon. (**B**) Macroscopic appearance of the cecum. (**C**) Weight of cecum contents. (**D**) pH of cecum contents. Results are expressed as mean ± SE. Significant differences between two groups at ** *p* < 0.01; *** *p* < 0.001. Cont, control (*n* = 5); VAN, vancomycin (*n* = 6); PB, polymyxin B (*n* = 5).

Colon length did not differ among the control and vancomycin- and polymyxin B-treated mice (Figure 3A). Macroscopic observation showed that the cecum was enlarged in vancomycin-treated mice, as reported previously [11], but not in polymyxin B-treated mice (Figure 3B). Indeed, the weight of the cecum content was significantly greater in the vancomycin-treated mice than in the controls (*p* < 0.001), whereas it did not differ significantly between polymyxin B-treated mice and the controls (Figure 3C). The pH of the cecum content was significantly higher in vancomycin-treated mice than in the controls or polymyxin B-treated mice (*p* < 0.01) (Figure 3D). On the other hand, there was no difference in pH between polymyxin B-treated mice and controls.

### 2.3. Effect of Antibiotic Treatment on Intestinal Permeability Ex Vivo

**Figure 4:**
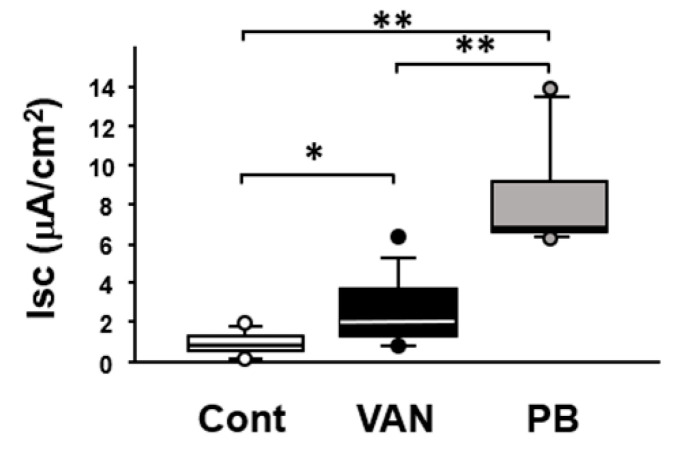
Effect of antibiotic treatment on intestinal permeability in mice. Macromolecular permeability was assessed using the Ussing chamber system. Data are presented as medians and interquartile ranges. Cont, control (*n* = 8); VAN, vancomycin (*n* = 9); PB, polymyxin B (*n* = 6). Significant differences between two groups at * *p* < 0.05; ** *p* < 0.01.

After treatment with the antibiotics for 7 days, macromolecular permeability was measured using the Ussing chamber system. The baseline short-circuit current (Isc) was significantly greater in the colon of vancomycin- and polymyxin B-treated mice than in that of the controls under the present experimental conditions (Figure 4).

### 2.4. Expression of Tight Junction Proteins in the Colon of Mice Treated with Antibiotics

**Figure 5:**
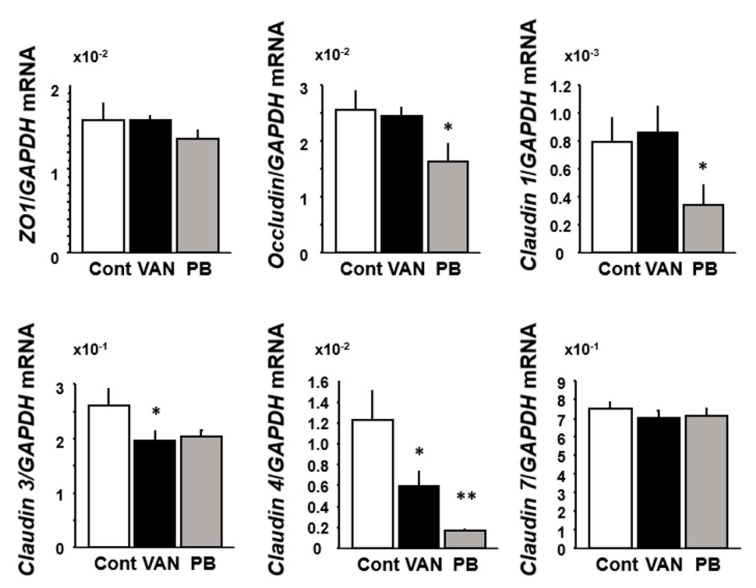
Effect of antibiotic treatment on the mRNA expression of tight junction molecules in mice colonic tissues. Results are expressed as mean ± SE. Cont, control (*n* = 5); VAN, vancomycin (*n* = 6); PB, polymyxin B (*n* = 5). Significantly lower than in the control: * *p* < 0.05, ** *p* < 0.01.

We investigated the expression of TJ-associated genes in the colonic tissues. Among those genes, expression of *claudin 3* and *claudin 4* mRNA was significantly decreased in vancomycin-treated mice relative to the controls. In polymyxin B-treated-mice, expression of *occuludin*, *claudin 1*, and *claudin 4* mRNA was significantly decreased relative to the controls (Figure 5). 

**Figure 6:**
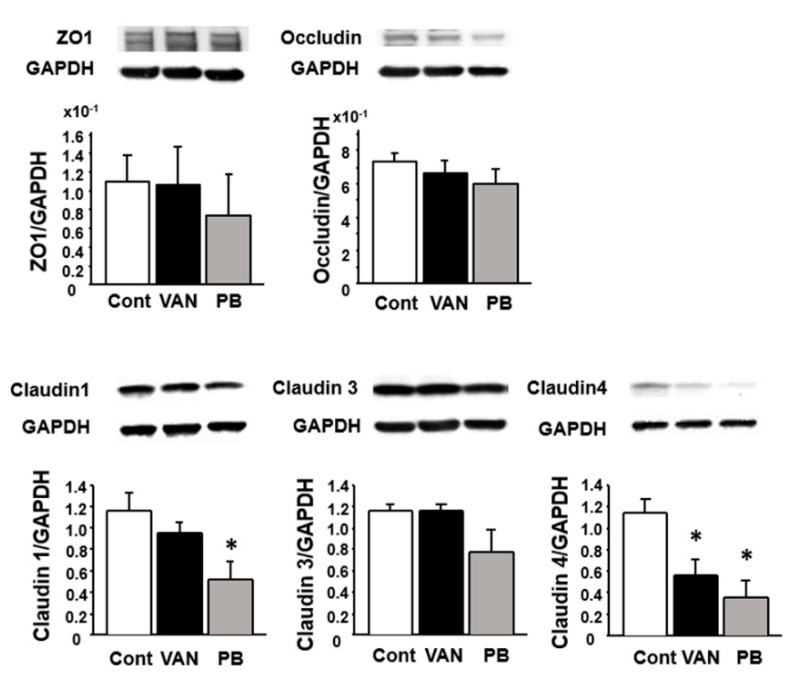
Effect of antibiotic treatment on the expression of tight junction proteins in mice colonic **tissues.** Results are expressed as mean ± SE (*n* = 5 in each group). Cont, control; VAN, vancomycin; PB, polymyxin B. Significantly lower than in the control: * *p* < 0.05.

We further investigated the expression of TJ-associated molecules at the protein level by Western blotting. The expression of claudin 1 was decreased in the colon of polymyxin B-treated mice (*p* < 0.05), and claudin 4 expression was significantly decreased in both vancomycin- and polymyxin B-treated mice relative to the controls (Figure 6).

**Figure 7:**
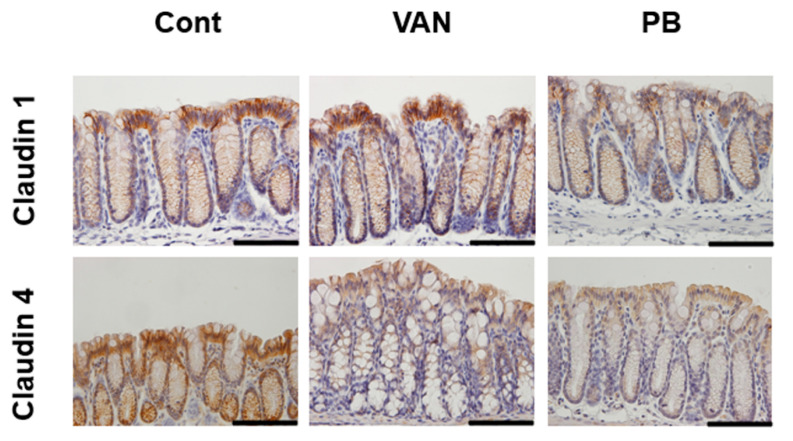
Immunostaining of claudin 1 and claudin 4 in the colonic mucosa of mice treated with vancomycin or polymyxin B. Cont, control; VAN, vancomycin; PB, polymyxin B. Bars = 100 μm.

Moreover, we examined the localization of immunoreactivity for tight junction proteins in the colonic mucosa of the experimental mice (Figure 7). The immunoreactivity of claudin 1 and claudin 4 was observed mainly on the luminal side of the colonic crypts. The intensity of claudin 4 immunoreactivity was apparently weaker in both vancomycin- and polymyxin B-treated mice relative to the controls; that of claudin 1 was weaker in polymyxin B-treated mice than in the controls. The immunoreactivity of other TJ proteins was also detected on the luminal side of the colonic crypts; however, there were no evident differences between the controls and vancomycin- or polymyxin B-treated mice (data not shown). 

### 2.5. Expression of Cytokines in the Colon of Mice Treated with Antibiotics

**Figure 8:**
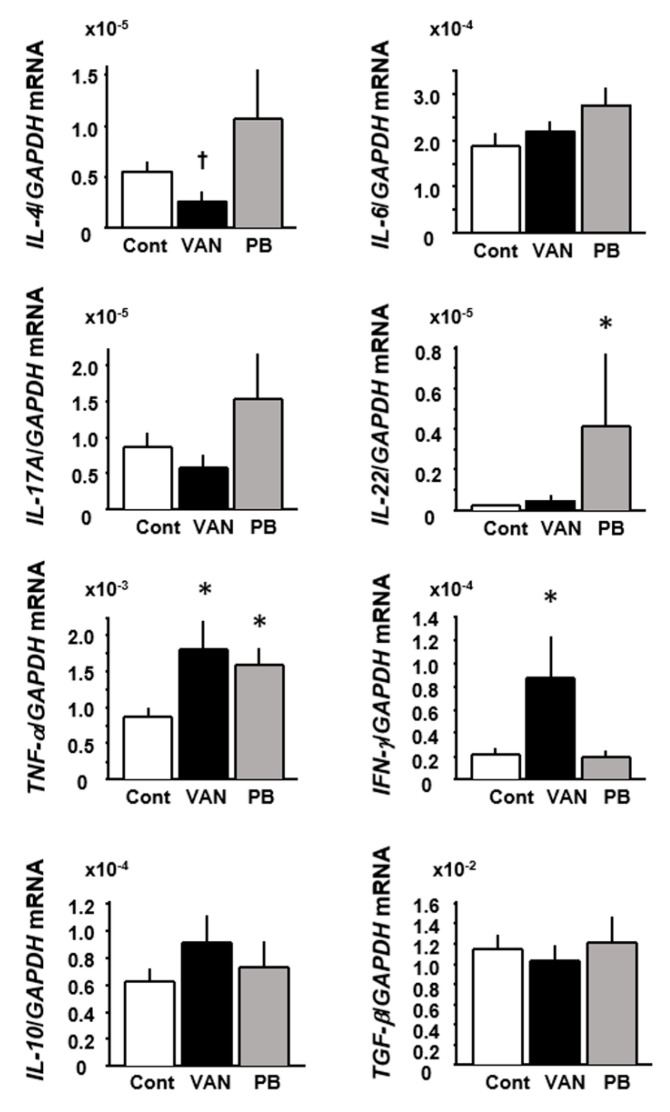
Effect of antibiotic treatment on the expression of cytokine mRNA in mouse colonic tissues. Results are expressed as mean ± SE. Cont, control (*n* = 5); VAN, vancomycin (*n* = 6); PB, polymyxin B (*n* = 5). Significantly higher than in the control: * *p* < 0.05. Significantly lower than in the control: † *p* < 0.05.

We next examined the gene expression of cytokines in the colonic tissue of the experimental mice (Figure 8). Compared to the control mice, the expression of *IL-4* was significantly decreased in vancomycin-treated mice (*p* < 0.05), whereas the expression of *IFN-γ* and *TNF-α* was increased in those mice relative to the controls (*p* < 0.05). In the colonic tissues of polymyxin B-treated mice, the expression level of *TNF-α* and *IL-22* was significantly increased compared to that in the control group (*p* < 0.05) (Figure 8).

### 2.6. Effect of the Cytokines IFN-γ and TNF-α on Intestinal Permeability In Vitro

**Figure 9:**
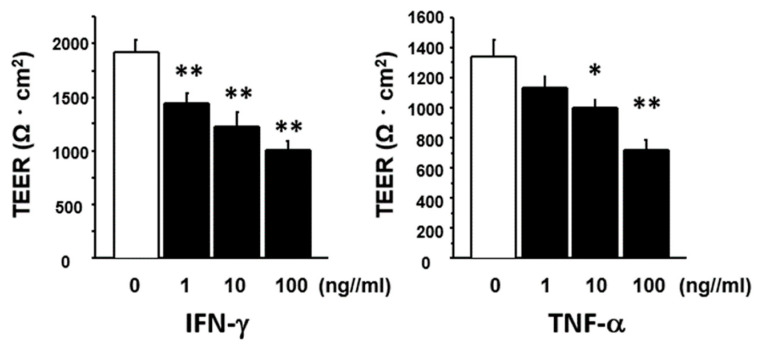
Effect of IFN-γ and TNF-α treatment on transepithelial electrical resistance (TEER). Caco2 cells (2 × 10^4^) were plated on 24-well culture inserts and cultured for 10 days. The cells were then stimulated with recombinant human IFN-γ (*n* = 6) or TNF-α (*n* = 5) at varying concentrations for 48 h. Results are expressed as mean ± SE. Significantly lower than the level in untreated controls; * *p* < 0.05, ** *p* < 0.01.

As the expression of *IFN-γ* and/or *TNF-α* was increased in the colon of vancomycin-treated or polymyxin B-treated mice, we next examined the effect of IFN-γ and TNF-α on the permeability of the intestinal epithelial cell layer in vitro. As shown in Figure 9, stimulation with both IFN-γ and TNF-α significantly and dose-dependently decreased the level of transepithelial electric resistance (TEER) in the Caco2 cell layer.

## 3. Discussion

Accumulating evidence has suggested that alteration in the gut microbiota is associated with the development of metabolic, psychological, and gastrointestinal functional disorders [11,12,13,14]. However, it has remained largely unknown how gut dysbiosis is involved in the pathophysiology in these disorders. It has been hypothesized that an imbalance in the gut microbiota disturbs mucosal barrier function in the gastrointestinal tract, making the gut more permeable to invading pathogens and leading to host inflammatory reactions [1,15]. To investigate the effect of dysbiosis on mucosal barrier function and the immune system, we treated mice with two different antibiotics and showed that their gut microbiome profiles became very different from those of normal control mice. Furthermore, as expected, vancomycin- and polymyxin B-treated mice also differed in their gut microbiome profiles, as the two different antibiotics attack Gram-positive and -negative bacteria, respectively. The diversity of the gut microbiome was clearly more decreased in vancomycin-treated than in polymyxin B-treated mice under the present experimental conditions. When analyzing the gut microbiome at phylum and genus levels, several differences were detected between not only control and antibiotic-treated mice but also between vancomycin- and polymyxin B-treated mice (Figure 2). These findings suggested that it would be possible to prepare two kinds of dysbiosis models.

Subsequently, we observed the colon of the experimental mice macroscopically and found that the cecum was apparently enlarged in the vancomycin-treated mice, but not in the polymyxin B-treated mice. In addition, we found that the cecum contents had a significantly higher pH than was the case in the control and polymyxin B-treated mice. These findings suggested a significant alteration of the luminal environment of the colon, at least in the vancomycin-treated mice. Interestingly, it has been reported that the amount of SCFAs or secondary bile acids is decreased in the colon of rats treated with vancomycin [16,17]. Moreover, it was noteworthy that the permeability of the colon was significantly elevated in both the vancomycin-treated and polymyxin B-treated mice. Thus, it has been clearly shown that vancomycin-induced dysbiosis is closely linked to alteration of the luminal environment and accelerated permeability of the colon.

To examine the molecular mechanism underlying the alteration of colonic mucosal permeability, we investigated the expression of colonic TJ proteins in the experimental mice, as TJ proteins play a pivotal role in the maintenance of mucosal barrier function. Screening of TJ proteins and their mRNAs showed that the expression of several TJ molecules was reduced in vancomycin- and/or polymyxin B-treated mice at both the mRNA and/or protein level. It is difficult to conclude which molecule plays a critical role in alteration of mucosal permeability in our dysbiosis model. However, reduction in claudin 4 expression may play a role in the acceleration of colonic mucosal permeability, as claudin 4 expression was clearly reduced in the colon of both vancomycin- and polymyxin B-treated mice, as revealed by not only Western blotting but also immunohistochemistry. 

In the present study, we were able to show that the acceleration of mucosal permeability was accompanied by the reduced expression of claudin 4 protein and other TJ molecule mRNAs in the colon under dysbiotic conditions. As to the factors affecting mucosal permeability and TJ protein expression in the colonic epithelium, it is tempting to speculate that the gut microbiome affects colonic epithelial cells directly, or indirectly via their metabolites [18]. For example, possible pathogens such as *Escherichia* were increased in vancomycin-treated mice. On the other hand, it is noteworthy that the pH of the cecum contents was significantly increased in vancomycin-treated mice, being compatible with a previous study [16]. Interestingly, it has been reported that the levels of SCFAs, especially butyrate, are decreased in the cecum feces of rats treated with vancomycin [16], suggesting that a similar alteration might have occurred in our vancomycin-treated mice. SCFAs are important metabolites produced by the gut microbiome, and are capable of stimulating colonic epithelial cells via G protein-coupled receptors [19], thus likely affecting mucosal barrier function [20,21]. Thus, although we were unable to measure the level of SCFAs in the colon, mucosal barrier parameters such as permeability might be affected by alterations in the colonic luminal environment, including gut contents and their microbiome profile.

We also investigated alterations in the cytokine profiles of colonic tissues in the dysbiotic mice. We had initially expected that the cytokine profiles would differ between vancomycin- and polymyxin B-treated mice because two types of dysbiosis induced by these antibiotics are quite different in view of the bacterial strains they target. However, as shown in Figure 8, we found no evident differences in colonic cytokine expression between vancomycin- and polymyxin B-treated mice. As a whole, *TNF-α* expression was increased in both sets of dysbiotic mice relative to the controls, and increased expression of *INF-γ* and *IL-22* was characteristic of vancomycin- and polymyxin B-treated mice, respectively. These data suggest that acceleration of proinflammatory cytokine expression may be commonly induced in the colon under any type of dysbiosis condition. However, it remains unsolved whether enhanced expression of proinflammatory cytokines is a cause or result of accelerated mucosal permeability. In this regard, we tested whether TNF-α and IFN-γ stimulation was able to accelerate the permeability of epithelial cell layers in vitro and showed that this was indeed the case. On the other hand, it can also be envisaged that pathogen invasion due to accelerated permeability of the colonic mucosa would activate immune cells to produce proinflammatory cytokines. Thus, the complex interrelationship existing among dysbiosis, accelerated mucosal permeability, and activation of the immune system in the colon remains a difficult issue to resolve. 

In summary, the treatment of mice with antibiotics was shown to cause dysbiosis accompanied by an acceleration in gut permeability and decrease in TJ protein expression in the colonic mucosa. Furthermore, we showed that the expression of proinflammatory cytokines such as *TNF-α* and *IFN-γ* was increased in colonic tissues under dysbiotic conditions, and that stimulation with these cytokines enhanced the permeability of layered colonic epithelial cells *in vitro*. Although the interrelationships of dysbiosis, accelerated mucosal permeability, and immune system activation in the colon still remain to be clarified, the present findings at least suggest that antibiotic-induced dysbiosis is linked to acceleration of gut permeability and subsequent and/or casual enhancement in proinflammatory cytokine expression in colonic tissues. As probiotics treatment is known to improve dysbiosis, we next plan to investigate whether probiotics treatment suppresses the enhanced mucosal permeability in the vancomycin/polymyxin B-induced dysbiotic mice. 

## 4. Materials and Methods 

### 4.1. Antibiotic Treatments

Specific pathogen-free mice (ICR, 6 weeks old, male) were obtained from Clea Japan (Tokyo, Japan) and used for the following experiments. To create dysbiotic conditions in the gut, mice were orally administered vancomycin (0.1 mg/mL; Sigma, Saint Louis, MO, USA) or polymyxin B (0.7 mg/mL; Sigma) in drinking water for 7 days, whereas controls were supplied with untreated water [16,22]. The colonic tissues were then obtained from the experimental mice, and the following parameters were determined: Length of the colon, weight of the cecum, and pH of the cecum contents. 

The obtained colonic tissues were transferred to Krebs buffer for the Ussing chamber assay. They were cut open along the longitudinal axis and fixed in neutral aqueous phosphate-buffered 10% formalin for histological evaluation, or stored in liquid nitrogen for real-time RT-PCR and Western blot analyses. The experimental protocol was approved by the Animal Use and Care Committee at Hyogo College of Medicine (No. 18-005, 29 May 2018).

### 4.2. Real-Time RT-PCR

Total RNA was isolated from the colonic tissues with TRIzol reagent (Invitrogen, Waltham, MA, USA). Total RNA (4 µg) was reverse-transcribed using an oligo (dT) primer (Applied Biosystems, Branchburg, NJ, USA), and real-time RT-PCR was carried out using a 7900H Fast Real-Time PCR System (Applied Biosystems) as described previously [23]. The primers used are shown in Supplementary Table S1. Real-time RT-PCR assays were performed with 200 ng of RNA-equivalent cDNA, SYBR-Green Master Mix (Applied Biosystems), and 500 nmol/L gene-specific primers. The PCR cycling conditions were 50 °C for 15 s and 60 °C for 60 s. The intensity of the fluorescent dye was determined, and the expression levels of target gene mRNA were normalized to the expression level of *glyceraldehydes-3-phosphate dehydrogenase* (*GAPDH*) mRNA.

### 4.3. Western Blot Analysis

Proteins were extracted from whole colonic tissue as described previously [24]. The protein extract (30 µg) was fractionated by sodium dodecyl sulfate polyacrylamide gel electrophoresis and transferred to a polyvinylidene difluoride membrane. The membrane was blocked with 5% (*w*/*v*) milk in PBS-T buffer for 1 h at room temperature and then incubated with the following specific primary antibodies: Rabbit anti-ZO-1 (dilution; 1:500, Invitrogen, Camarillo, CA, USA), rabbit anti-occludin (dilution; 1:250, Invitrogen), rabbit anti-claudin 1 (dilution; 1:500, Invitrogen), rabbit anti-claudin 3 (dilution; 1:1000, Invitrogen), rabbit anti-claudin 4 (dilution; 1:250, Invitrogen), and rabbit anti-GAPDH (dilution; 1:1000, Cell Signaling Technology, Beverly, MA, USA) at 4 °C overnight. After washing with PBS-T buffer, the membranes were incubated for 1 h with the corresponding horseradish-peroxidase-conjugated secondary antibodies (Cell Signaling Technology). The target proteins were detected using an enhanced chemiluminescence system (Amersham Biosciences, Buckinghamshire, UK). ImageJ software (NIH) was used for quantifying the intensities of the target bands. The staining intensity of GAPDH was set as the internal control. The values in individual tests were expressed as fold values of the target protein/GAPDH in the standard group.

### 4.4. Immunohistochemistry

Immunohistochemical staining was performed with an Envision Kit (Dako, Kyoto, Japan) in accordance with the manufacturer’s protocol using the following antibodies: Anti-ZO-1 (dilution; 1:200, Invitrogen, Camarillo, CA, USA), anti-Occludin (dilution; 1:50, Invitrogen), anti-claudin 1 (dilution; 1:400, Invitrogen), anti-claudin 3 (dilution; 1:400, Invitrogen), and anti-claudin 4 (dilution 1:200; Abcam, Cambridge, UK). In brief, the sections were deparaffinized, rehydrated, and treated by microwave heating for 20 min in Antigen Unmasking Solution (Vector Laboratories, CA, USA) as described previously with minor modifications [25]. To quench endogenous peroxidase activity, the sections were preincubated with 0.3% H_2_O_2_ in methanol for 20 min at room temperature, and then incubated with the primary antibodies for 60 min at room temperature. Thereafter, the slides were washed in PBS, incubated with horseradish peroxidase-conjugated secondary antibody for 30 min, visualized using 3,3′-diaminobenzidine tetrahydrochloride with 0.05% H_2_O_2_ for 3 min, and then counterstained with Mayer’s hematoxylin. 

### 4.5. Ussing Chamber Assay

The Ussing chamber assay was performed as described previously [12]. In brief, segments of the colon were cut along the mesenteric border and mounted in Ussing chambers (Physiologic Instruments, San Diego, CA, USA) to expose a tissue area of 0.3 cm^2^ to circulating oxygenated Krebs buffer (115 mM NaCl, 1.25 mM CaCl_2_, 1.2 mM MgCl_2_, 2.0 mM KH_2_PO_4_, 25 mM NaHCO_3_) at 37 °C. Mannitol (10 mM) and glucose (10 mM) were added to the Krebs buffer for the mucosal and serosal sides, respectively. Thereafter, short-circuit current (Isc) values were obtained at equilibrium as described previously [26] about 20 min after the tissues had been mounted, and expressed as μA/cm^2^.

### 4.6. Measurement of Transepithelial Electrical Resistance

The human intestinal epithelial cell line Caco2 was cultured in RPMI 1640 medium (Sigma, Saint Louis, MO, USA) with 10% fetal bovine serum (Gibco, NY, USA) and 1% penicillin/streptomycin (Sciencell, San Diego, CA, USA) in a humidified incubator at 37 °C with an atmosphere of 5% CO_2_. Caco2 cells were seeded and grown to confluence on 24-well culture inserts (0.4 µm pore size; Corning, NY) and then stimulated in the absence or presence of various concentrations of recombinant human IFN-γ or TNF-α (R&D Systems, Minneapolis, MN, USA) for 48 h in the basolateral chamber.

Electrical resistance across the stratified epithelium was measured using a Millicell-ERS-2 instrument (Millipore, Bedford, MA, USA) with “chopstick’’ electrodes, as described previously [24]. The value obtained from a blank insert was subtracted to give the net resistance, which was then multiplied by the membrane area to give the resistance in area-corrected units (Ω·cm^2^). Transepithelial electrical resistance (TEER) values were recorded 48 h after stimulation.

### 4.7. Extraction of DNA from Fecal Samples

Extraction of bacterial DNA was performed as described previously [12,27]. In brief, the fresh fecal samples were resuspended in a solution containing 450 µL of extraction buffer (100 mM Tris-HCl, 40 mM EDTA; pH 9.0) and 50 µL of 10% sodium dodecyl sulfate. Then, 300 µg of glass beads (diameter, 0.1 mm) and 500 µL of buffer-saturated phenol were added to the suspension, vortexed vigorously, and centrifugated at 14,000× *g* for 5 min, and 400 µL of the supernatant was collected. The DNA was eluted from the supernatant by the phenol-chloroform method.

### 4.8. Illumina Library Generation and DNA Sequencing

Analysis of the 16S rDNA of the fecal microbiome was performed according to a method described previously [28] with minor modifications. Briefly, the V3-V4 region of 16S rDNA was amplified using the primers, as reported previously [28], and then ligated with overhang Illumina adapter consensus sequences. The PCR was performed once at 95 °C for 3 min and then for 25 cycles at 95 °C for 30 s, 55 °C for 30 s, and 72 °C for 30 s, and finally at 72 °C for 5 min on a Veriti thermal cycler (Thermo Fisher Scientific, Waltham, MA, USA). The amplicon was purified using AMPure XP magnetic beads (Beckman Coulter, Brea, CA, USA). The Illumina Nextera XT Index kit (Illumina, San Diego, CA, USA) with dual 8-base indices was used to allow for multiplexing. To incorporate two unique indices to the 16S amplicons, PCR reactions were performed as described previously [11,12]. The libraries were purified using AMPure XP beads, quantified fluorometrically using a QuantiT PicoGreen ds DNA Assay Kit (Invitrogen, Paisley, UK), and then diluted to 4 nM using 10 mM Tris-HCl (pH 8.0), followed by pooling of the same volume for multiplex sequencing. The multiplexed library pool (10 pM) was spiked with 40% PhiX control DNA (10 pM) to improve base calling during sequencing. Sequencing was conducted using a 2 × 250-bp paired-end run on a MiSeq platform with MiSeq Reagent Kit v2 chemistry (Illumina).

### 4.9. DNA Sequence Analysis

Demultiplexing and removal of indices were performed using the MiSeq Reporter software (Illumina) as reported previously [8]. Filtering out of low-quality sequences, removal of chimera sequences, construction of operational taxonomic units (OTUs), and taxonomy assignment were performed using the Quantitative Insights into Microbial Ecology (QIIME) pipeline (http://qiime.org/) [29]. Thereafter, 30,000 raw reads were randomly obtained from the sequence files for each sample and merged by fastq-join with the default setting. Sequence reads with an average quality value of <25 were removed, and then chimera-checked. Five thousand high-quality sequence reads were randomly obtained for each sample, and OTUs for total high-quality reads were constructed by clustering with a 97% identity threshold. The representative reads of each OTU were then assigned to the 16S rRNA gene database using UCLUST with ≥97% identity. Comparison of each taxon in the gut microbiota was conducted at both phylum and genus level. The Shannon and Chao 1 indices were calculated to examine the alpha diversity of the microbiota in the samples.

### 4.10. Statistical Analysis

All values were expressed as means ± SE. For analyses of gut microbiota, statistical significance was determined by Welch’s *t*-test with Benjamini–Hochberg correlation. The significance of differences between two animal groups was analyzed by the Mann–Whitney *U*-test. Differences were considered to be significant at *p* < 0.05. 

## Supplementary Materials

Supplementary materials can be found at https://www.mdpi.com/1422-0067/21/17/6108/s1. Table S1: Primers for reverse transcription-polymerase chain reaction analysis.

## Abbreviations

TJ	tight junction
PCoA	principal coordinate analysis
TEER	transepithelial electric resistance
OTU	operational taxonomic unit
SCFA	short-chain fatty acid
PBS	phosphate buffered saline
GAPDH	glyceraldehydes-3-phosphate dehydrogenase
