# Supplementary material for: Alteration of Colonic Mucosal Permeability during Antibiotic-Induced Dysbiosis
Source: Int J Mol Sci. 2020 Aug 25;21(17):6108. doi: 10.3390/ijms21176108 (PMC7504080; doi:10.3390/ijms21176108)
Supplement: Supplementary file 1 [file ijms-21-06108-s001.pdf]

Table S1. Primers for reverse transcription-polymerase chain reaction analysis

Genes	Direction	Sequences
IL-4	Forward	5'-GAATGTACCAGGAGCGATATC-3
	Reverse	5’-CTCAGTACTACGAGTAATCCA-3’
IL-6	Forward	5’-CCAGTTGCCTTCTTGGGACT-3’
	Reverse	5’-CCAGTTGCCTTCTTGGGACT-3’
IL-10	Forward	5’-TGGACAACATACTGCTAACCG-3’
	Reverse	5’-GGATCATTTCCGATAAGGCT-3’
IL-17A	Forward	5’-GACTCTCCACCGCAATG-3’
	Reverse	5’-CGGGTCTCTGTTTAGGCT-3’
IL-22	Forward	5’-TCCGAGGAGTCAGTGCTAA-3’
	Reverse	5’-AGAACGTCTTCCAGGGTGAA-3’
IFN-γ	Forward	5’-GCATCTTGGCTTTGCAGCT-3’
	Reverse	5’-CCTTTTTCGCCTTGCTGTTG-3’
TNF-α	Forward	5’-GGTGCCATGTCTCAGCCTCTT-3’
	Reverse	5’-GCCATAGAACTGATGAGAGGGAG-3’
TGF-β	Forward	5’-GCTGAACCAAGGAGACGGAAT-3’
	Reverse	5’-GCTGATCCCGTTGATTTCCA-3’
ZO-1	Forward	5’-TGCAATTCCAAATCCAAACC-3’
	Reverse	5’-AGAGACAAGATGTCCGCCAG-3’
Occludin	Forward	5’-TTGAAAGTCCACCTCCTTACAGA-3’
	Reverse	5’-CCGGATAAAAAGAGTACGCTGG-3’
Claudin1	Forward	5’-TTAGTGGCCACAGCATGGTA-3’
	Reverse	5’-GAAGGTGTTGGCTTGGGATA-3’
Claudin3	Forward	5’-CCAACTGGGTACAAGACGAG-3’
	Reverse	5’-TCTTGGTGGGTGCATACTTG-3’
Claudin4	Forward	5’-GGAGGGCCTCTGGATGAACT-3’
	Reverse	5’-GATGCTGATGACCATAAGGGC-3’
Claudin7	Forward	5’-AGCATGTTCCTGGATTGGTC-3’
	Reverse	5’-CCAGAAGGACCAGAGCAGAC-3’
GAPDH	Forward	5’-GGAGAAACCTGCCAAGTATG-3’
	Reverse	5’-TGGGAGTTGCTGTTGAAGTC-3’

IL, interleukin; *IFN-γ*, interferon-γ; *TNF-α*, tumor necrosis factor-α; *TGF-β*, transforming growth factor-β; *ZO-1*, Zona occludens-1.
